# Distributed Refractive Index Sensing Based on Etched Ge-Doped SMF in Optical Frequency Domain Reflectometry

**DOI:** 10.3390/s23094361

**Published:** 2023-04-28

**Authors:** Cailing Fu, Ronglong Sui, Zhenwei Peng, Yanjie Meng, Huajian Zhong, Mingquan Li, Xiaoyu Yin, Yiping Wang

**Affiliations:** 1 Shenzhen Key Laboratory of Photonic Devices and Sensing Systems for Internet of Things, Guangdong and Hong Kong Joint Research Centre for Optical Fiber Sensors, State Key Laboratory of Radio Frequency Heterogeneous Integration, Shenzhen University, Shenzhen 518060, China; 2 Shenzhen Key Laboratory of Ultrafast Laser Micro/Nano Manufacturing, Key Laboratory of Optoelectronic Devices and Systems of Ministry of Education/Guangdong Province, College of Physics and Optoelectronic Engineering, Shenzhen University, Shenzhen 518060, China; 3 Guangdong Laboratory of Artificial Intelligence and Digital Economy (SZ), Shenzhen 518107, China

**Keywords:** distributed fiber sensing, refractive index sensors, germanium-doped fibers, optical frequency domain reflection

## Abstract

A distributed optical fiber refractive index sensor based on etched Ge-doped SMF in optical frequency domain reflection (OFDR) was proposed and demonstrated. The etched Ge-doped SMF was obtained by only using wet-etching, i.e., hydrofluoric acid solution. The distributed refractive index sensing is achieved by measuring the spectral shift of the local RBS spectra using OFDR. The sensing length of 10 cm and the spatial resolution of 5.25 mm are achieved in the experiment. The refractive index sensing range is as wide as 1.33–1.44 refractive index units (RIU), where the average sensitivity was about 757 GHz/RIU. Moreover, the maximum sensitivity of 2396.9 GHZ/RIU is obtained between 1.43 and 1.44 RIU.

## 1. Introduction

The optical fiber refractive index sensor has aroused great interest in chemical and biological applications due to its advantages: compact size, high sensitivity, electromagnetic interference, and real-time sensing [1,2]. To date, various optical fiber refractive index sensors have been proposed and developed to achieve high sensitivity based on tilted fiber grating [3,4,5], fiber Bragg grating [6], the Fabry–Perot interferometer [7,8,9], the Mach–Zehnder interferometer [10,11,12], the D-shaped surface plasmon resonance [13], and the fluid-filled photonic crystal fiber coupler [14,15]. However, the aforementioned refractive index sensors are single-point sensors, which could not be used for the real-time monitoring of solution diffusion or the reaction process in safety checking of marine ships and quality inspection in chemical industry applications.

In recent years, distributed optical fiber sensors combining optical time domain, frequency domain, or correlation domain technologies with Rayleigh [16], Brillouin [17], and Raman scattering [18] in the fiber could be used to sense the basic parameters, such as temperature and strain [19]. To make the light propagating within the fiber core interact with the surrounding medium, namely, the refractive index sensor, various methods have been attempted. For example, Zadok et al., report an optomechanical fiber sensor based on forward stimulated Brillouin scattering by radial, guided modes of the single mode fiber (SMF) with no structural intervention to sense the surrounding medium [20]. Then, a 3-cm long side polished SMF was also proposed to measure the surrounding refractive index of the liquid, where the evanescent fields of pump and probe light waves involved in Brillouin sensing are free to interact with the surrounding environment. However, the sensing length and low refractive index sensitivity were limited by the side polished section, weak interaction between Brillouin scattering, and the evanescent field, respectively [21].

Rayleigh backscattering in the fiber is caused by the inhomogeneities’ refractive-index being distributed in the core, resulting from the uneven internal density during the fiber-drawing process. The Rayleigh scattering spectrum, like a fingerprint, is constant and randomly distributed once the fiber is fabricated. When the temperature or strain is applied to the fiber, the Rayleigh scattering spectrum shifted due to thermal and elastic optical effects. Consequently, various sensors using Rayleigh backscattering have been proposed and demonstrated. Since 1998, distributed temperature and strain sensors based on optical frequency domain reflectometry (OFDR) were proposed by recognizing the Rayleigh backscattering (RBS) between the measurement RBS and reference RBS with a cross-correlation operation [22]. Compared with a lower spatial resolution using Brillouin scattering technology, the OFDR technology based on RBS has a higher spatial resolution and sensitivity, i.e., the order of a millimeter [23]. Therefore, distributed sensors based on OFDR have been widely used in various fields, such as structural health monitoring and shape sensing. However, the Rayleigh scattering light propagating in the fiber core cannot penetrate the cladding, which limits the application to be a refractive index sensor. To achieve distributed refractive index sensing, various structures have been attempted to make the RBS limited in the fiber core interact with the surrounding refractive index. In 2017, Du et al., presented a distributed refractive index sensor by bending a SMF into a radius of curvature of several millimeters to make RBS leak into fiber cladding, where the sensitivity was 2319.24 GHz/RIU with a bending diameters of 12.2 mm [24]. However, only the curved part of this sensor could be used for refractive index sensing, and the RBS loss was introduced by bending. Thus, this refractive index sensor was still a point sensor, not a distributed sensor. In 2018, Ding et al., presented a distributed OFDR refractive index sensor based on a tapered optical fiber with a diameter of 4.3 μm, where the spatial resolution and measurement distance were 4.25 mm and 2.1 cm, respectively [25]. The sensor exhibited a high sensitivity attributed to the strong coupling between higher-order modes and evanescent fields. Meantime, a distributed refractive index sensor with a sensitivity of 1.53 nm/RIU was obtained by simply etching a MgO-nanoparticle-doped SMF with hydrofluoric acid (HF) [26]. However, the sensitivity and sensing length of this special customized fiber were limited by the weak interaction with the external medium. In 2021, another type of distributed refractive index sensor only in the curved part was realized by bending a SMF into a U-shape to excite sets of higher order modes [27]. Subsequently, a temperature compensated distributed refractive index sensor using an etched multi-core fiber was proposed, where an outer core and inner core were used for distributed refractive index sensing and temperature compensation, respectively [28]. Although the temperature change could be compensated by using a multi-core fiber, it is difficult to obtain an etched core with the same size by etching. Therefore, to achieve a distributed refractive index sensing, it is necessary to use bending, tapering, and etching technology to radiate the light wave propagating in the fiber core out of the fiber cladding.

In this letter, a distributed refractive index sensor using etched Ge-doped SMF in optical frequency domain reflectometry was proposed and demonstrated. The Rayleigh backscattering (RBS) amplitude of the Ge-doped SMF is 5.0 dB higher than that of standard SMF. The etched Ge-doped SMF was obtained by only using wet-etching, i.e., hydrofluoric acid solution. The surrounding refractive index was detected by measuring the spectral shift of the local RBS spectra using OFDR. The refractive index measurable range is 1.33–1.44 RIU, and the maximum sensitivity is 2396.9 GHz/RIU when the surrounding refractive index was increased from 1.43 to 1.44. Moreover, the length of the sensing fiber is 10 cm, where the spatial resolution is 5.25 mm. The loss property of the Ge-doped SMF induced by etching was investigated. The refractive index sensing property of the etched Ge-doped SMF under different residual cladding diameters was simulated and experimentally investigated.

## 2. Materials and Methods

**Figure 1:**
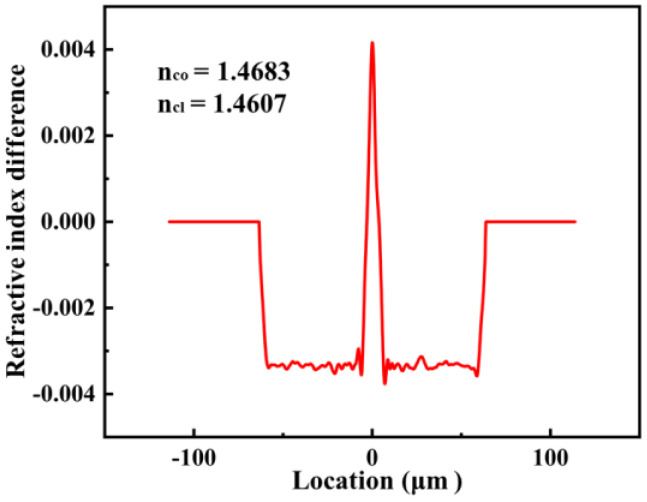
The refractive index difference diagrams of the Ge-doped SMF, where the refractive index of the core and cladding are 1.4683 and 1.4607, i.e., *n_co_* = 1.4683, *n*_cl_ = 1.4607, respectively. Note that the matching oil with a refractive index of 1.464 was used as the substrate for the refractive index measurement.

However, a higher spatial resolution and accuracy were limited by the inherent weak RS signal in the conventional optical fiber, i.e., single mode fiber (SMF). Although various methods, such as the UV laser and femtosecond laser, have been employed to enhance the intensity of Rayleigh scattering in fiber, these methods are relatively expensive and complex. To achieve a long sensing length and high spatial resolution, a commercially available Ge-doped SMF (F-SM-1500, Newport) with a higher Rayleigh scattering was employed to replace the standard SMF. The core and cladding diameter were 8.4 and 125 μm, respectively, whose size was the same as the standard SMF. The refractive index distribution of Ge-doped SMF was measured by a 3D reconstruction system refractive index (SHR-1802, Shanghai University), where the working wavelength is 632.8 nm. The refractive index distribution of the fiber could be calculated based on the best digital holography obtained from the measured phase projection distribution by using angular spectrum theory and the filtered back projection algorithm. Note that the matching oil with a refractive index of 1.464 was used as the substrate for the refractive index measurement. As shown in Figure 1, the core and cladding refractive index difference were 0.0043 and −0.0033, respectively, corresponding to the core and cladding refractive index of 1.4683 and 1.4607 at the wavelength of 632.8 nm, respectively. In addition, the difference in refractive index under different working wavelengths in the simulation could be ignored. Therefore, the core and cladding at the wavelength of 1570 nm, i.e., the working wavelength in the experiment, are also considered to be 1.4683 and 1.4607, respectively.

Before the Ge-doped SMF was etched, the evanescent field of the core could not interact with the surrounding medium, i.e., it was insensitive to the surrounding refractive index. To achieve the distributed surrounding refractive index sensing, the Ge-doped SMF should be etched until its core could interact with the surrounding medium. A part of light guided in the core of etched Ge-doped SMF is coupled into the cladding and generates high-order modes. These high-order modes propagating in the cladding have larger evanescent waves, which penetrate the surrounding medium. Then, the high-order modes interacting with the surrounding medium are recoupled to the core and interfered with the fundamental mode transmitted in the core [25]. The principle of refractive sensing is that the change in the surrounding refractive index causes change in the evanescent field, which in turn causes change in the effective refractive index of the etched optical fiber. In this way, the variation of the refractive index of the surrounding medium could be detected.

**Figure 2:**
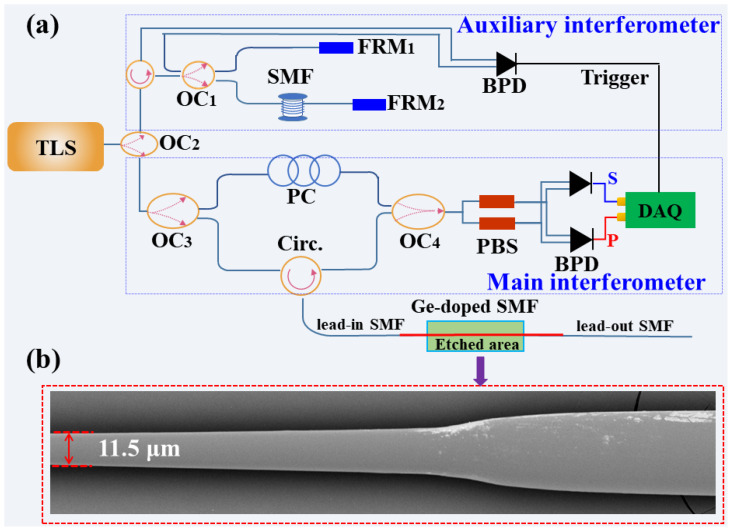
(**a**) Schematic diagram of the distributed refractive index sensing using etched Ge-doped SMF based on optical frequency domain reflectometry (OFDR); (**b**) Scanning electron microscopy of etched Ge-doped SMF with hydrofluoric acid (HF), where the remained diameter of the etched Ge-doped SMF is 11.5 μm. TLS: tunable laser source; OC: optical coupler; PC: polarization controller; DF: delay fiber; FRM: faraday rotating mirror; BPD: balanced photo-detector; PBS: polarization beam splitter; CIR: circulator; DAQ: data acquisition card.

The hydrofluoric (HF) acid was used to etch the Ge-doped SMF. The experiment was conducted in a constant temperature fume hood due to the danger of the HF acid, where the etching device was made of polytetrafluoroethylene material. The fabrication process of the etched Ge-doped SMF was listed as follows. Firstly, the coating of the Ge-doped SMF was removed by immersing in acetone for 40 min instead of fiber stripper to avoid any damage on the fiber surface. Secondly, the Ge-doped SMF without coating was immersed in HF acid solution with a concentration of 30% and 5% for 135 min and 15 min, respectively, to obtain an etched Ge-doped SMF with a uniform diameter and smooth surface. Finally, the etched Ge-doped SMF was cleaned with deionized water and 10% sodium hydroxide solution to remove the remaining HF acid. As shown in Figure 2b, the etched Ge-doped SMF obtained by the wet-etching method has a smooth surface and uniform diameter through a scanning electron microscope.

## 3. Experiments and Results

An experimental setup for distributed refractive index sensing based on OFDR was illustrated in Figure 2a. The demodulated principle of OFDR is optical coherence detection, which has significant characteristics such as a large dynamic range, high spatial resolution, and high sensitivity. The light from the tunable laser source (TLS) was split into two paths by a 10:90 optical coupler (OC_2_), where the scanning range of the TLS was 1530–1610 nm, respectively. The 10% light was injected into an auxiliary interferometer with a Michelson structure, where the generated signal was used as the external clock of data acquisition card (DAQ) to sample the beat signal from the balanced photo-detector (BPD) at equidistant instantaneous optical frequency points. Two Faraday rotating mirrors (FRMs), i.e., FRM_1_ and FRM_2_, make the auxiliary interferometer insensitive to the polarization state of light. The 90% light was injected into the main interferometer, a Mach-Zehnder structure, and split by a 50:50 OC_3_. Then, the Rayleigh back-scattering (RBS) reflected by the fiber under test (FUT) was mixed with the reference light passing through the polarization controller (PC) by the OC_4_, where the PC was used to adjust the polarization state. Two polarization beam splitters (PSBs) were employed to divide the acquired signal into p- and s-polarization to reduce the polarization fading. The measurement path of the main interferometer was connected to the Ge-doped SMF through lead-in SMF, where the other end was also spliced with the lead-out SMF. The initial size of the Ge-doped SMF is the same as that of lead-in/out SMF. Finally, the beat frequency signals of the measurement and reference are collected by BPD and converted into electrical signals through a high-speed acquisition card. The principle of the refractive index sensing is that the wavelength of the RBS spectrum measured by the OFDR system in Figure 1 would shift with the change of the refractive index of the surrounding medium.

**Figure 3:**
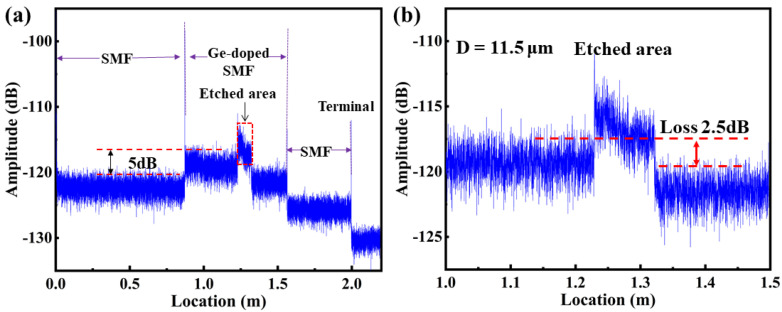
(**a**) Measured Rayleigh backscattering (RBS) amplitude in the spatial domain along the FUT, containing lead-in SMF, Ge-doped SMF, and lead-out SMF, respectively, where the average amplitude of the Ge-doped SMF was 5 dB higher than that of lead-in SMF; (**b**) Enlarged view of the measured RBS amplitude under the residual cladding diameter of 11.5 μm, where an overall loss before and after etching was 2.5 dB.

As we know, the etched Ge-doped SMF could be used to interact with the surrounding medium, that is, for refractive index sensing, only when the core was etched to have loss. In the experiment, the loss pattern was monitored by the OFDR system illustrated in Figure 2a during the etching process. In this way, the cladding diameter of Ge-doped SMF was etched to 11.5 μm, i.e., the residual cladding diameter of the etched Ge-doped SMF was 11.5 μm, where the etched length was about 10 cm, as illustrated in Figure 2b. In addition, the length of lead-in SMF, Ge-doped SMF and lead-out SMF was approximately 87, 69, and 43 cm, respectively, as illustrated in Figure 3a. The average Rayleigh back-scattering (RBS) amplitude of the Ge-doped SMF was 117.35 dB, which is 5.0 dB higher than that of lead-in SMF, i.e., 122.35 dB. Moreover, the average amplitude of the etched area was much higher than that of the unetched area, resulting from the Fresnel reflection at medium-cladding interface. In addition, it was observed that an overall 2.5 dB loss before and after etching was not perfectly constant, attributed to the uneven diameter in each point of the etched area. It should be noted that the 2.5 dB loss after etching was measured at an external refractive index of 1.33.

**Figure 4:**
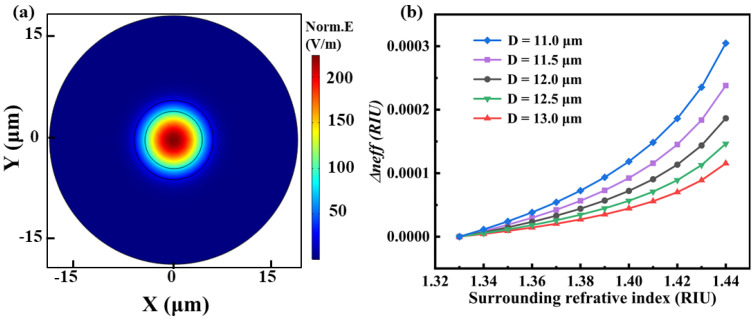
(**a**) Simulated fundamental mode in etched Ge-doped SMF; (**b**) Simulated effective mode index, i.e., $\Delta n_{eff}$, as a function of the surrounding refractive index, where the residual diameter of the etched Ge-doped SMF was decreased from 13.0 to 11.0 μm with a step of 0.5 μm. Note that the $\Delta n_{eff}$ was increased exponentially with the increase of the surrounding refractive index.

To investigate the refractive index sensing property of the etched Ge-doped SMF, the effective mode index, i.e., $\Delta n_{eff}$, was firstly simulated by use of COMSOL Multiphysics under residual cladding diameter of 13.0, 12.5, 12.0, 11.5, and 11.0 μm, respectively. The simulated fundamental mode of the etched Ge-doped SMF was illustrated in Figure 4a, where three concentric circles with different diameters represent the fiber core, remained cladding, and external refractive index sample. As shown in Figure 4b, the $\Delta n_{eff}$ of the etched Ge-doped SMF was increased exponentially, when the surrounding refractive index was increased from 1.33 to 1.44; the stepper of the parametric sweep is 0.01. Moreover, the variation in $\Delta n_{eff}$ was increased with the decrease of the residual cladding diameter of the Ge-doped SMF, indicating that the refractive index sensitivity was increased with the decrease of the residual cladding diameter. However, the loss would increase with the decrease of the residual cladding diameter, resulting in the reduction of the signal to noise (SNR) near the etched end. Therefore, the Ge-doped SMF was etched to 11.5 μm, i.e., D = 11.5 μm, in consideration of the balance between the sensing distance and refractive sensitivity, as illustrated in Figure 3b.

A series of liquid samples with different refractive indices used to investigate the refractive index sensing property were prepared by mixing glycerol and distilled water with different concentrations, where the refractive index was measured and calibrated at 25 °C. The etched Ge-doped SMF with a residual diameter of 11.5 μm was immersed in refractive index liquid samples with different refractive index. After each test, the sample was cleaned carefully by the use of alcohol to remove the residual refractive index liquids on the surface of fiber. The spectral shift induced by surrounding refractive index could be obtained by the cross-correlation of the RBS.

The detailed processing procedure for refractive index sensing was listed as follows. Firstly, the reference and measurement signal with different surrounding refractive index were collected separately. Note that the previous surrounding refractive index, i.e., 1.33, was used as the reference surrounding refractive index to demodulate the next surrounding refractive index, i.e., 1.34, and so on. Secondly, fast Fourier transform (FFT) was conducted to transform reference and measurement signal from frequency domain to the spatial domain; the information of each reflection point on the optical fiber will be displayed, as shown in Figure 3a. Thirdly, a sliding window with a width of ΔX that contains *N* data points was used to select the local RBS, i.e., *N* = 500. The smaller the value of *N*, the higher the spatial resolution, the lower the signal to noise ratio of the cross-correlation signal, and the greater the spectral drift error. Generally, each sliding window was padded with zeros to $2^{M}$ and transformed back to frequency domain through inverse FFT (IFFT). The signal processing program is the same as the RBS based OFDR distributed temperature and strain sensing. Thus, the sensing spatial resolution, i.e., Z, could be given by

(1) Z=NΔz.

**Figure 5:**
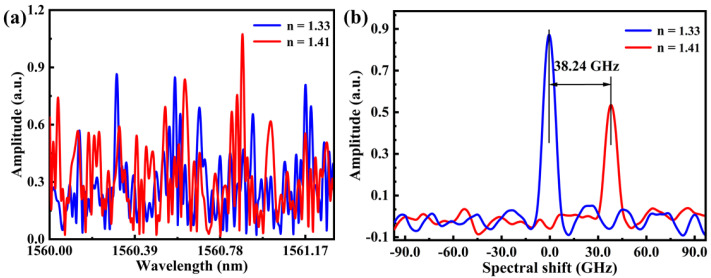
(**a**) Local RBS spectrum at surrounding refractive index of 1.33 and 1.41, labeled by blue and red curve, respectively. (**b**) Cross-correlation of two different local RBS spectra, where the spectral shift was 38.24 GHz.

The two-point spatial resolution, i.e., Δz, in distance domain could be given by

(2) $$\Delta z=\frac{c}{2n\Delta F},$$

where *c* is the light velocity in vacuum, *n* is the refractive index of the medium, ∆*F* is the range of the sweep frequency of the TLS, i.e., ΔF=9736 GHz. In the experiment, the sensing spatial resolution was 10.5 μm, i.e., *Z* = 0.525 cm. Finally, the cross-correlation operation between local reference and measurement signal were performed to obtain the spectral shift, where the refractive index variation of the surrounding refractive index could be obtained by the spectral shift of the local RBS. As shown in Figure 5a, the local Rayleigh scattering spectra at surrounding refractive index of 1.33 and 1.41 were labeled by blue and red curve, respectively. As shown in Figure 5b, the spectral shift of 38.24 GHz could be obtained by cross-correlation, when the surrounding refractive index was changed from 1.33 to 1.41. Compared with the cross-correlation peak at refractive index of 1.33, the cross-correlation peak at refractive index of 1.41 was still significant, but its amplitude decreased. This indicated that the cross-correlation peak would deteriorate with the increase of the refractive index.

**Figure 6:**
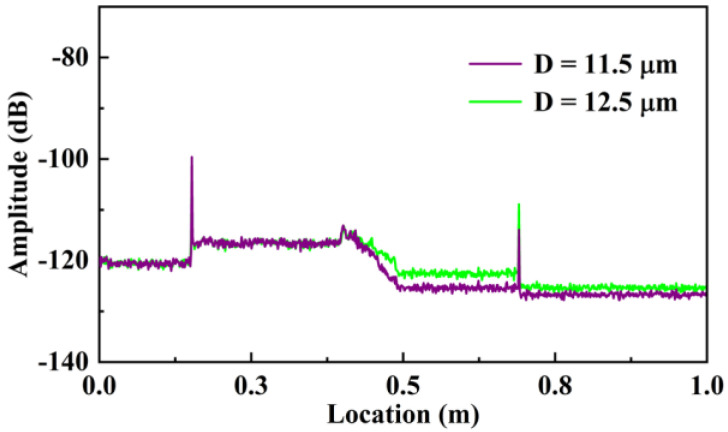
Measured Rayleigh backscattering (RBS) amplitudes of the etched Ge-doped SMF with residual diameters of 11.5 and 12.5 μm under the surrounding refractive index of 1.44, labeled by purple and green curve, respectively.

To investigate the effect of the residual diameter on the surrounding refractive index sensitivity and sensing spatial resolution, two types of etched Ge-doped SMF with residual diameters of 11.5 and 12.5 μm were employed. Firstly, the loss property of the etched Ge-doped SMF was studied. As shown in Figure 6, the measured RBS amplitude of etched Ge-doped SMF with residual diameter of 11.5 μm was significantly greater than that of 12.5 μm. Moreover, the RBS amplitude at the end of the etched area was close to the bottom noise, i.e., −127.41 dB, under the surrounding refractive index of 1.44, when the Ge-doped SMF was etched to 11.5 μm. As we know, the distributed refractive index sensing was dependent on the loss of the RBS. In addition, the sensing length and refractive index sensitivity were mutually restricted. When the standard SMF was etched to 11.5 μm, the amplitude of the RBS could not maintain the loss of evanescent waves, resulting in the inability to demodulate the spectral shift of the RBS, i.e., the inability to demodulate external refractive index changes. Therefore, the Ge-doped SMF with a higher RBS amplitude than standard SMF was employed.

**Figure 7:**
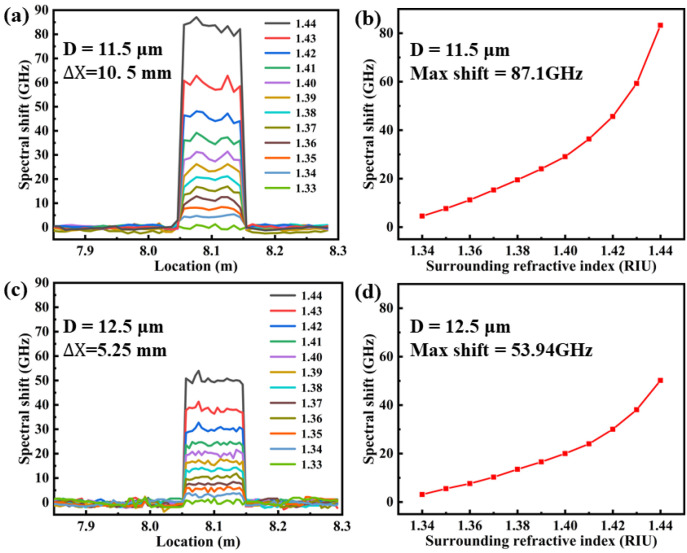
Measured spectral shift of the RBS at the residual diameters of etched Ge-doped SMF of (**a**,**b**) 11.5 μm and (**c**,**d**) 12.5 μm, respectively, as a function of surrounding refractive index, while the refractive index was increased from 1.33 to 1.44 with a step of 0.01.

Moreover, the distributed refractive index sensing property was further compared and studied at a temperature of 25 °C, when the surrounding refractive index was increased from 1.33 to 1.44. In the experiment, we found that the loss of RBS increases as the refractive index of the external environment increases. Note that the sensing range was limited between 1.33 and 1.44 due to RBS amplitude of 127.41 dB at refractive index of 1.44, as illustrated in Figure 6. The spectral shift of the etched area was measured at different surrounding refractive index, ranging from 8.05 to 8.15 m, i.e., a length of 10 cm. Note that the sensing spatial resolution is 10.5 mm and 5.25 mm, respectively, for etched Ge-doped SMF with residual diameters of 11.5 and 12.5 μm, respectively, which indicates that there are 10 and 20 continuous effective sensing points in the length of 10 cm, i.e., etched area. As shown in Figure 7a,c, the fluctuation of the spectral shift at different sensing points was observed, resulting from the incompletely smooth etched surface. As shown in Figure 7b,d, the average spectral shift was increased exponentially with the increase of surrounding refractive index, which was in good agreement with the simulation in Figure 4. Obviously, the max spectral shift was 87.1 GHz and 53.94 GHz, when the surrounding refractive index was increased from 1.33 to 1.44, respectively. Therefore, the average sensitivity is about 757 GHz/RIU and 456.4 GHz/RIU over the entire sensing range. Moreover, the surrounding refractive index sensitivity was up to 2396.9 and 1209.1 GHz/RIU, when the surrounding refractive index was increased from 1.43 to 1.44, corresponding to the spectral shift of 23.9 and 12.1 GHz. Compared with the simulated results in Figure 4, the obtained sensitivity in the experiment was higher. The reason is that only the effective refractive index change of the fundamental mode is considered in the simulation. In fact, the effective refractive index changes of the high-order modes transmitted in the cladding were the main reasons for the RBS shift. Compared with Refs. [24,25,26,27], the advantage of the proposed sensor, i.e., etched Ge-doped SMF, is that the sensing length could be achieved up to 10 cm. The obtained refractive index sensitivity and sensing length could be further improved by using fiber with high RBS amplitude.

## 4. Conclusions

We have proposed and demonstrated a distributed refractive index sensor using etched Ge-doped SMF in optical frequency domain reflectometry. The etched Ge-doped SMF was obtained by only using wet-etching, i.e., hydrofluoric acid solution. The refractive index measurable range is 1.33–1.44 RIU, where the average sensitivity was about 757 GHz/RIU. In addition, the maximum sensitivity of 2396.9 GHZ/RIU is obtained between 1.43 and 1.44 RIU. Moreover, the length of the sensing fiber is 10 cm, where the spatial resolution is 5.25 mm. The Rayleigh backscattering (RBS) amplitude of the Ge-doped SMF is 5.0 dB higher than that of standard SMF, and an overall 2.5 dB loss was induced by the etching process. The refractive index sensitivity was increased with the decrease of the residual cladding diameter. To balance the loss and refractive index sensitivity, an etched Ge-doped SMF with a residual diameter of 11.5 μm was obtained. The surrounding refractive index was demodulated by measuring the spectral shift of the local RBS spectra using OFDR. A fiber with stronger RBS has a potential to achieve a longer sensing distance and higher refractive index sensitivity, such as using RBS-enhanced fiber obtained through UV laser exposure or femtosecond laser microfabrication methods. In addition, the cross sensitivity induced by temperature has not been considered, and the temperature compensation mechanism could be introduced to improve the performance of the proposed sensor.

## Data Availability

The data presented in this study are available on request from the corresponding author.
